# Cryosurgery Vs Trichloroacetic Acid Chemical Cautery for the Treatment of Hypertrophied Nasal Turbinate: A Comparative Study

**DOI:** 10.22038/ijorl.2019.39039.2293

**Published:** 2020-09

**Authors:** Satish S. Satpute, Samir V. Joshi, Ripudaman Arora, Neel Prabha, Prashant Keche, Nitin M. Nagarkar

**Affiliations:** 1 *Department of Otorhinolaryngology and Head and Neck Surgery, All India Institute of Medical Sciences, * *Raipur, Chhattisgarh, * *India.*; 2 *Department of Otorhinolaryngology and Head and Neck Surgery, BJ Government Medical College, Pune, India.*; 3 *Department of Dermatology, All India Institute of Medical Sciences, Raipur, Chhattisgarh, India.*; 4 *Department of Otorhinolaryngology and Head and Neck Surgery, SVN Govt Medical College, Yavatmal, India.*

**Keywords:** Cryosurgery, Inferior turbinate hypertrophy, Trichloroacetic acid

## Abstract

**Introduction:**

The Study Aimed To Compare The Results of Cryosurgery With Trichloroacetic Acid Chemical (TCA) Cautery For The Treatment of Hypertrophied Nasal Turbinates.

**Materials and Methods:**

This was a prospective study of 70 patients with nasal obstruction due to hypertrophied nasal turbinates who were randomly divided in 2 groups of 35 each. In group 1, patients underwent inferior nasal turbinate reduction by cryosurgery and in group 2 patients with cautery by 50 % TCA. The patients were evaluated using SNOT-20 score pre and postoperatively on 6th month.

**Results:**

Significant improvement in symptoms was seen in 28 patients (80 %) in group 1 and in 10 patients (28.57%) in group 2..Improvement in average SNOT Score after cauterization of hypertrophied nasal turbinates by cryosurgery was from 55(severe) to 16(mild) and by TCA was from 54(severe) to 32(mod) in 6 months and this was statistically significant (P<0.001). Complications like bleeding, scarring, infection and adhesion formation were more with TCA than that of cryosurgery group and among these complications scarring was statistically significant (P =0.003).

**Conclusion:**

The use of cryosurgical treatment for hypertrophied nasal turbinates is a safe, curative method as compared to that of TCA cautery which is less curative and with more complications.

## Introduction

Chronic nasal obstruction is a very common distressing symptom in the world which is often the reason for otolaryngological consultation. There are multiple etiological factors which may lead to a periodical or permanent nasal obstruction. The main causes of this problem are inferior turbinate hypertrophy and septal deformities.^1^Hypertrophied inferior nasal turbinate are usually found in many conditions like perennial allergic rhinitis and vasomotor (non-allergic) rhinitis. Turbinate enlargement in these patients is usually bilateral and is caused by a thickening of the mucosa without hypertrophy of the underlined structures (1).

 Nasal obstruction due to hypertrophied nasal inferior turbinates is medically treated by antihistamines, topical/systemic decongestant, intranasal corticosteroid sprays and immunotherapy. The patients not responding to medical treatment are treated with surgical approaches like injection of corticosteroids or sclerosing agents (2), turbinectomy and turbinoplasty, cryosurgery, laser ablation surgery, lateral fracture of the turbinate, sub mucosal ablation by electrocautery and chemical cauterization (1).

 Chemical cauterization is an old established technique for temporary symptomatic relief, while cryosurgery is comparatively a new technique for long term symptomatic relief in allergic rhinitis. It is not clear which of these two procedures offer better results with few complications. Accordingly, we conducted a study to compare the results of cryosurgery with trichloroacetic acid (TCA) chemical cautery for the treatment of hypertrophied nasal turbinates.

## Materials and Methods

A prospective, randomized controlled clinical study was carried out in department of Otorhinolaryngology, Head and Neck Surgery between October 2008 and December 2010 after obtaining permission from Institutional Ethical Committee. 

Seventy patients of chronic nasal obstruction due to hypertrophied inferior nasal turbinates were included in this study after taking informed written consent and Inclusion criteria was clinically diagnosed cases of bilateral chronic nasal obstruction due to hypertrophied inferior nasal turbinates with SNOT Score of 40- 60 and disease free nasopharynx who were unresponsive to medical management of age range 20-40 years (3). Patients having active sinus infection, nasal polyps, hypertrophied adenoids, previous nasal surgery and with immunocompromised status were excluded from the study. All patients underwent a detailed history, clinical examination, hearing (tuning fork tests and pure tone audiometry) and nasal endoscopic examination, blood (complete hemogram, liver and renal function tests, HIV, hepatitis B and C serology) and urine analysis. Detailed nose examination with anterior rhinoscopy and pre-operative nasal endoscopy was done to rule out any other nasal pathology and all exclusion criteria. Post-operative nasal endoscopy was done to analyze the effect of treatment on nasal turbinates. The patients were randomly assigned into two treatment group. Each group consists of 35 patients. In group 1 patients underwent inferior nasal turbinate reduction by cryosurgery and in group 2 patients underwent cautery by 50% trichloroacetic acid. Patient’ symptoms were subjectively analyzed with the help of SNOT SCORE preoperatively and postoperatively on 1st month, 3rd month and 6th month.

**Sino Nasal Outcome Test- 20 (SNOT- 20) Score: **The SNOT- 20 is a modified version of the 31-Item Rhinosinusitis Outcome Measure (RSOM- 31), and it contains 20 nose, sinus, and general items. Patients describe their disease-specific health status by indicating the severity of rhinosinusitis symptoms and describe their Quality of Life by indicating importance across different domains, including the physical problems, functional limitations, and emotional consequences of rhinosinusitis (3). For scoring of SNOT score and grading of improvement after 6 months the following scale was used:

0–20 mild, significant improvement; 20–40 moderate, partial improvement; 40–60 severe, no improvement both the procedures were performed on an out-patient basis in minor surgery room. 

Topical anesthesia was obtained by packing both nostrils with cotton gauge soaked in a solution of 4% xylocaine: adrenaline; 1:100000. In group 1 after removing xylocaine packs from the nostrils, a large flat, silver nasal cryoprobe was used. 

The nasal probe was covered medially to prevent inadvertent freezing of nasal septum, which could lead to subsequent perforation. 

The anatomical areas treated by cryotherapy were the lateral surface, the inferomedial and superomedial surface of the inferior turbinate and the medial surface of the middle turbinate. Particular care was taken to avoid the contact with middle meatus. (The instruments used for cryosurgery was Gemi cryosurgery unit manufactured by Glatron Medicure Ltd. Mumbai). In group 2 after removing xylocaine packs from the nostrils, 50 % TCA was taken on a cotton swab stick and then applied on inferomedial and superomedial surface of inferior turbinate. Particular care was taken to avoid contact to external skin and septum which can lead to scarring or synechiae formation. 

After either of this procedure the patients were observed in recovery room for 4 to 6 hours. In post- operative period, oral antibiotic, antihistamines, analgesics and topical nasal decongestant drops were given to every patient. Patients were followed on next day to see for any bleeding or scarring. Then patients were followed up postoperatively. Preoperative and 6^th^ month SNOT score were compared for final analysis of results.

**Statistical analysis**

The data analysis was done with the help of statistical software, SPSS Version 16. The data was summarized using descriptive statistics, frequencies and percentage. Statistical differences between categorical variables were assessed using the Chi-square test. A p-value <0.05 was considered statistically significant.

## Results

Out of total 70 patients included in study 52 (74.29%) were males and 18 (25.71%) were females. 

 Twenty-six (37.14%) patients were above 30 years and 44 (62.85%) patients were below 30 years of age. All the patients included in our study had bilateral nasal obstruction as a presenting complaint. Other complaints were headache in 65 (92.86%), followed by rhinorrhea in 62 (88.57%), post nasal discharge in 59 (84.28%) and sneezing in 55 (78.57%) patients. Symptom wise relief with treatment in each group is given in table 1. Symptomatic improvement was more in cryosurgery group as compare to TCA group and the difference was statistically significant. Figure 1 shows pre and postoperative photograph of a patient of group 1, inferior turbinate reduction was seen at 6 month follow up with cryosurgery.

**Fig 1a:**
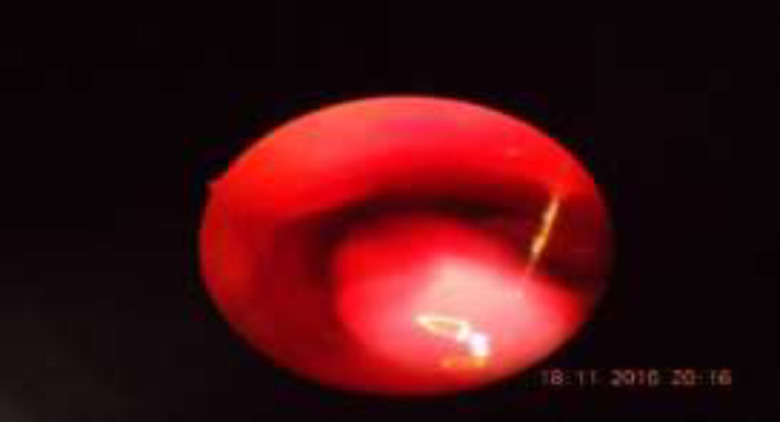
Hypertrophied left inferior turbinate preoperative

**Fig 1b:**
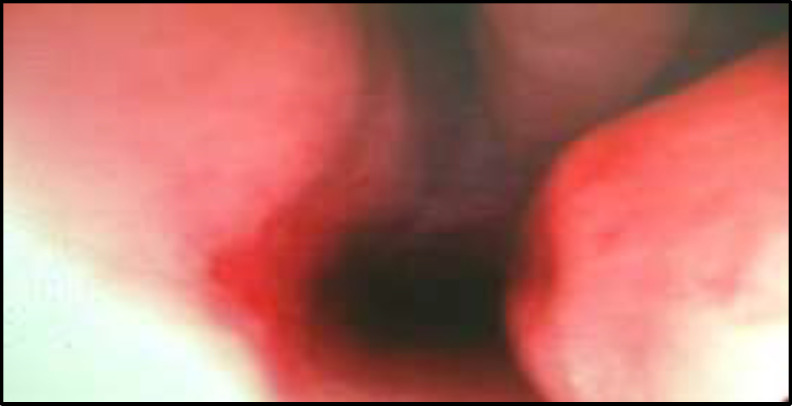
Inferior turbinate of same patient showing reduction in size at 6 month follow up with cryosurgery

**Table 1:** Symptom-wise relief after cauterization of nasal turbinate

**Sr. No.**	**Symptoms**	**Group 1**			**Group 2**			
		**No. of cases**	**No. of cases relieved**	**Percentage**	**No. of cases**	**No. of cases relieved**	**Percentage**	**p-value**
1	Nasal obstruction	35	28	80.00%	35	10	28.57%	<0.001
2	Rhinorrhea	32	26	81.25%	30	11	36.67%	<0.001
3	Sneezing	28	23	82.14%	27	9	33.33%	<0.001
4	Post nasal discharge	29	22	75.86%	30	12	40%	0.007
5	Headache	34	27	79.41%	31	9	29.03%	<0.001

In cryosurgery group all 35 patients had pre-operative SNOT score in the range of 40- 60 (Severe). After cryosurgery, 28 (80 %) patients had SNOT Score reduced to less than 20 (Mild), suggestive of clinically significant improvement and 5 (14.29%) patients had partial improvement with SNOT score reduced to range of 20-40 (Moderate). Thus 33 (94.28%) patients showed improvement (significant/partial) after cryosurgery. In TCA group all 35 patients had pre-operative SNOT Score in the range of 40- 60 (Severe). After TCA application 10(28.57%) patients had SNOT Score reduced to less than 20 (Mild), suggestive of clinically significant improvement and 20(57.14%) had partial improvement with SNOT Score reduced to range of 20-40 (Moderate). Thus 30 (85.71%) patients showed improvement (significant/ partial) after TCA application. 

There was not much difference in the total number of patients relieved in both groups (cryosurgery group - 33 patients, TCA group B- 30 patients), however 80% patients in cryosurgery group had their SNOT score dropped to less than 20 compared to only 28.57% patients of TCA group. Improvement in average SNOT Score after cauterization of hypertrophied nasal turbinates by cryosurgery was from 55(severe) to 16(mild) and by TCA was from 54(severe) to 32(mild) in 6 months (Table.2). 

**Table 2:** Average SNOT score after cauterization of hypertrophied nasal turbinates

**Average SNOT Score**							
**Group 1**				**Group 2**			
Pre-op	On follow up			Pre-op	On follow up		
	After 1 month	After 3 month	After 6 month		After 1 month	After 3 month	After 6 month
55	30	22	16	54	45	37	32
Severe	Moderate	Moderate	Mild	Severe	Severe	Moderate	Moderate

**Fig 2:**
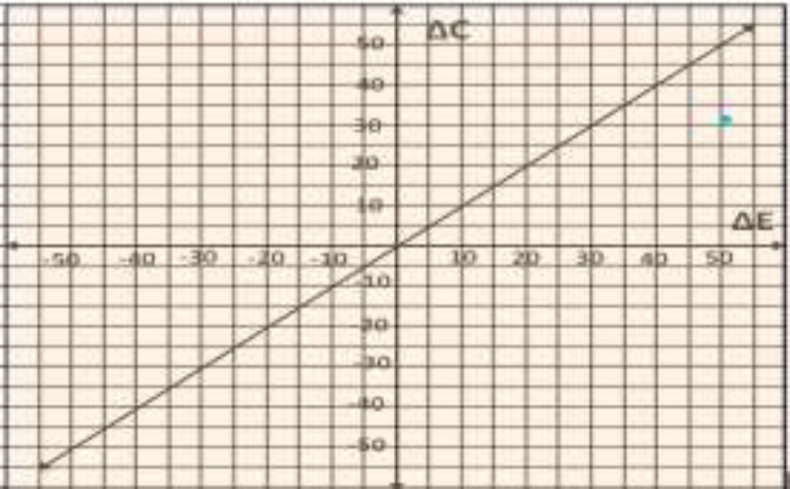
Cost-effectiveness of Cryosurgery as compared to TCA application

There was statistically significant improvement (P<0.001) in cryosurgery group than TCA group on the basis of SNOT score. Cost effectiveness analysis of cryosurgery and TCA cautery at the end of 6^th ^month is given in table 3 and figure 2.The blue dot in figure 2 shows that cryosurgery falls in the acceptability zone (C= 33.01 INR; E= 51.43).Therefore, we can conclude that cryosurgery was more cost effective than TCA application. Here we would like to mention that we have not included the cost of cryosurgery unit because this cryosurgery unit was being used in our institute 1 year before this study and according to manufacturer’s opinion it will be used for next 4 to 5 years, so till then lot more thanthe number of patients of this study will undergo cryosurgery treatment for turbinate hypertrophy. (The cost of cryosurgery unit was 17,438 INR, 604 USD). 

**Table 3:** Cost-effectiveness analysis of the turbinate cauterization at the end of 6^th^ month

**Parameters**	**Cryosurgery**	**TCA application**
Cost in INR/USD for 35 participants	1000/ 14.02	27/0.38
Cure rate (%) (effectiveness)	80.00% i.e. 28 patients	28.57% i.e. 10 patients
Cost effectiveness	1000 INR for 28 patients	27 INR for 10 patients
Cost needed to treat one case successfully (INR/USD)	35.71/0.5	2.7/0.03

Complications of both procedures are mentioned in table 4. Complications like bleeding, crusting, infection and adhesion formation were more with TCA than that of cryosurgery group and among these complications crusting was statistically significant (P=0.003). Figure 3 and 4 shows crusting after TCA application on 2nd day and adhesion formation with TCA at 6 month follow up respectively.

**Fig 3 a:**
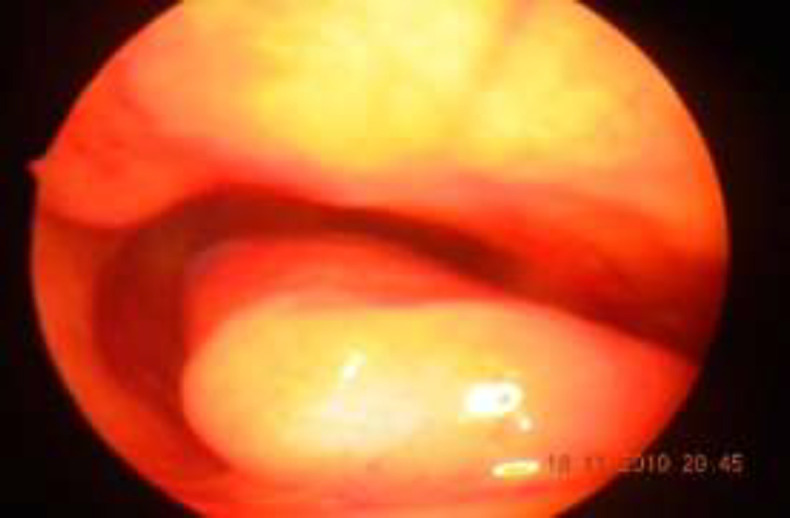
Hypertrophied right inferior turbinate preoperative

**Fig 3 b:**
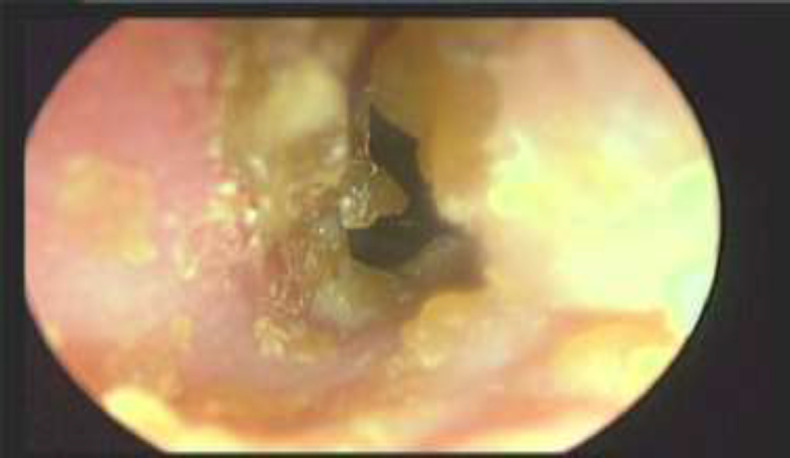
Crusting after TCA application on 2nd day

**Fig 4:**
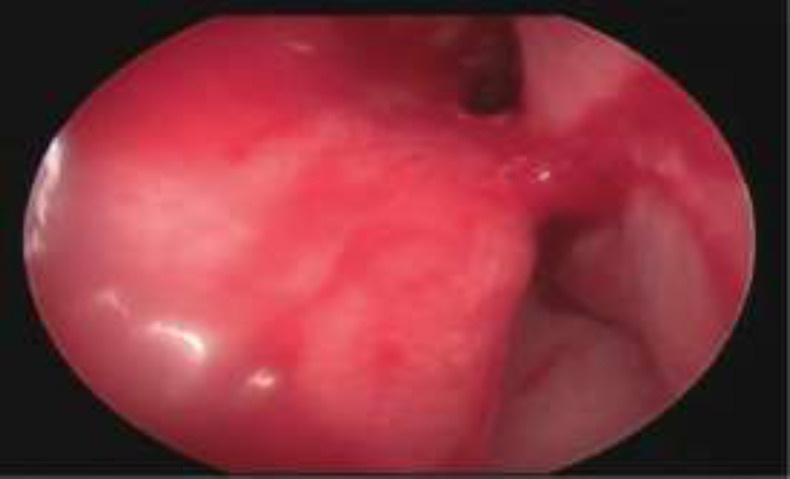
Adhesion formation with TCA at 6 month follow up

**Table 4:** Complications of cryosurgery and TCA application

**Sr. no.**	**Complication**	**Group 1**		**Group 2**		**P-value**
		**No. of cases**	**Percentage**	**No. of cases**	**Percentage**	
1	Bleeding	6	17.14%	13	37.14%	0.059
2	Crusting	4	11.42%	15	42.85%	0.003
3	Infection	3	8.57%	7	20%	0.172
4	Adhesion formation	7	20%	5	14.28%	0.527

## Discussion

Cryosurgery for chronic rhinitis was first introduced by Ozenberger (4). In this method under local anaesthesia with the help of cryoprobe cooling agent (nitrous oxide or liquid nitrogen) is applied on the surface of turbinates. There is intracellular ice crystals formation and protein denaturation and subsequent tissue destruction which lead to debulking of turbinates. The duration of application time varied from 5 seconds to 3 minutes (5,6). Kajra et al compared the results of different application time (15-20, 30-40, 60-120seconds) of cryosurgery in vasomotor and chronic persistent allergic rhinitis (7).

The results regarding nasal obstruction and increased secretion showed no statistical significant differences between different application times. The shortest application time (20-30 seconds) caused superficial mucosal necrosis and postoperative pain was less as compared to longer application time.

In literature different success rates of this method has been reported. Nasal obstruction was relieved in 52 to 92 % and rhinorrhea in 22 to 92% of patients in various studies (7-11). Ozenberger and Mehra et al reported significant improvement in sneezing (9,12).

In Varshney et al study it was relieved in only 26.9% patients (5). In our study nasal obstruction was relieved in 80%, rhinorrhea in 81% and sneezing in 82% of patients. Hartley et al in their study found that cryotherapy can provide long term benefit in patients with inferior turbinate hypertrophy even if minor septal deviation was present (13). However Brown et al and Yosephet al found that cryotherapy was effective for short term but effectiveness decreased with time (13,14). Puhakka et al suggested that cryotherapy results may improve with repeated application (15).

There is no major complications or sequelae of this procedure. Complications like epistaxis, nasal crusting, nasal adhesions, infection, ear blockage and scarring have been reported in various studies (5,10,15-18).

Principato noted bleeding during procedure in 10.6% of patients, but bleeding resolved with topical adrenaline (16). In our study, 17 % patients had bleeding during procedure with cryosurgery. In our study bleeding was only occasional and did not need any active intervention. Infection occurred in only 8.57% patients in our study. 

This complication was less than that of study conducted by Anand et al where infection occurred in 15% of patients (19). Adhesion formation was seen in 20% of our patients. Discomfort and pain were present in 14.29% of our patients for maximum 2 days duration. Chissone et al reported discomfort and pain in 23.4% patients and which persisted for 24 hr (11). Elwany et al study, discomfort and headache was seen in only 2% patients (20).

Trichloroacetic acid is an analog of acetic acid. TCA can be applied over mucosa or infiltrated in mucosa. Different concentrations of TCA has been used in various studies. The ideal concentration for nasal mucosa is not mentioned in the literature. 

Kazuo et al demonstrated that TCA treatment induced inhibition of Th 2 cell infiltration in nasal mucosa (21). This suggest that TCA treatment can inhibit local type 1 allergic reaction. TCA produce local astringent action by coagulating albumin. Sensitivity and excitability of nasal mucuosa reduces after TCA cautery (22). 

Unsal Tuna et al found that TCA treatment was effective for controlling nasal allergic symptoms without causing damage to the mucociliary function (23). Nemade et al compared efficacy of TCA chemical cautery, steroid nasal spray and combination of TCA cautery plus steroid nasal spray in the treatment of allergic rhinitis with inferior turbinate hypertrophy (24).At six months post treatment Symptom Severity Grade 1-2 was achieved in 45.4% patients in TCA group, 40.9 % in steroid nasal spray group and 70.4% in combined therapy group. Statistically significant improvement was found with combined treatment. 

In Honda et al study nasal obstruction was relieved in 77%, rhinorrhea in 85% and sneezing in 70% of patients with chemical cauterization of inferior turbinate by TCA (25). Anbaki et al found control of sneezing in 89.5 % and rhinorrhoea in 85.7% patients (26). However in our study nasal obstruction was relieved in 28%, rhinorrhea in 36% and sneezing in 33% of patients.

In a study by Anbaki et al and Nemade et al no obvious adverse effects of local application of 50 % TCA were seen (24,26). Azevedo et al reported pain, synechiae and epistaxis (27). 

In their study they infiltrated 30% TCA into nasal mucosa through insulin syringe. In our study 37.14% patients had post-operative bleeding and infection occurred in 20% patients. Purulent rhinorrhea which lasted for one week was also found in the study of Honda et al. (25) Adhesion formation was seen in 14.2%, severe irritation and pain in 19.2% of our patients. This was in accordance with the study by Honda et al where they also found severe to moderate degree of pain after TCA Application (25).

Both cryosurgery and TCA cautery are simple method and can be done in outpatient department. We compared the results of cryosurgery with TCA chemical cautery, we found cryosurgery to be more effective in controlling symptoms and more cost effective. Also, the complications were less with cryotherapy.

## Conclusion

Nasal obstruction is not a life-threatening condition but it disturbs all the routine activities of life. Nasal obstruction is mostly due to turbinate hypertrophy. This study included the patients with nasal obstruction due to hypertrophied inferior turbinates not relieving from conservative medical management. The use of cryosurgical treatment for hypertrophied turbinates is a safe, curative method as compared to that of chemical cauterization by TCA, which is less curative and with more complications. 
